# Observations on Milk-Sickness

**Published:** 1840-08

**Authors:** John Travis

**Affiliations:** Carroll County, Tennessee


*July*, 1840.

---

Art. V.—*Observations on Milk-Sickness.* By John Travis, M. D., of Carroll County, Tennessee.

Whenever a new disease occurs among mankind or the inferior animals, and its remote cause cannot be clearly demonstrated, the floodgates of imagination are thrown open, from which thousands of conjectures flow, some of which are of the most absurd and ridiculous character. Such was the case on the appearance of Asiatic cholera. It is the duty of the physician to examine, to write and to think of diseases, in a *rational* manner. It ill comports with the character of medical science in this enlightened age, to trace the origin of diseases to the influence of the sun, the moon, and the stars. I have as much faith in the judicial astrology of the Romans, or Hesiod's theory of the origin of the world, as I have in such notions.

That milk-sickness exists in certain localities, I cannot for a moment entertain a doubt. It has made its appearance annually in Carroll county, and some of the adjacent ones, since the first settlement of the Western District of Tennessee. Many instances of death among horned cattle have occurred in this county, and dogs feeding upon their dead carcases have become diseased, and some have died. My own dogs have had the disease in such a manner as to render them unable to walk, by feeding on a dead cow. Several persons of this vicinity have had the disease, and some have died of it. They have had the whole train of symptoms described by Professor Yandell in his essay on milk-sickness, in the Transylvania Journal of Medicine, vol. 1, No. 3. I have invariably pursued the course of practice suggested in that essay, in the management of the cases that have come under my care.

The great concern of the physician is to obviate the constipation that exists, which is often of the most obstinate character, and with the removal of which the nausea, vomiting, and other distressing symptoms generally yield. Whether the relief from cathartics be owing to the removal of the poison of milk-sickness from the primæ viæ, or to the restoration of the alvine secretions, is a matter not yet decided among practitioners, but of the efficacy of these remedies in the disease, no one who has had experience in it entertains a doubt. Venesection in the early stage is useful, not only in calming the symptoms, but in promoting the operation of the cathartic. The gastric irritation continuing after the use of the lancet and purgatives, a large blister is applied to the epigastrium. Sinapisms to the extremities are beneficial in equalizing excitement, and affusions of cold water answer a similar purpose when much heat of the surface is present. In a word, the treatment does not differ materially from that pursued in bilious fever, except that constipation being a more prominent symptom will require more attention. That being obviated, the disease in most cases becomes sufficiently manageable.

It may not be uninteresting to state a remarkable fact which has occurred under my own observation; it is, that my own cattle range with those of my neighbors, and have done so for five years, and none of them have ever had the disease, while many of theirs have died. I am strongly inclined to believe, that this exemption is owing to my giving my cattle salt every other day, during the spring, summer and autumnal months, whereas other cattle get salt perhaps once a month. From this fact it would appear, that the muriate of soda, (common salt,) acts as a prophylactic of the disease.

A range of hills of considerable elevation runs up from near my mills on Sandy river, westward, between which are fertile valleys. In these ravines cattle often range, and, it appears, also contract this disease. In these valleys abounds the Rhus radicans, upon which cattle sometimes feed. From certain experiments made, some years ago, by a gentleman near Cincinnati, there appears some reason to believe, that a small poison vine, a species of the *Rhus*, will cause milk-sickness. He had a number of cattle in a lot, in the winter season, where the vine grew; he observed them to eat of the vine, contract the disease, and die. He removed the vines and put other cattle in the same lot, which escaped the disease. This is the fairest experiment that I have ever known made, and it certainly should have considerable bearing upon this interesting and obscure subjéct. I intend at an early period to make experiments with the poison vine growing in this region, and will communicate the result to the Editors of the Western Journal.

I am aware, that great discrepancy exists among physicians respecting milk-sickness, some even denying its existence, *in toto*, and others deeming it nothing more than a form of *congestive fever*. It certainly bears a remote resemblance to congestive fever; but by certain diagnostic symptoms, pointed out in the essay to which I have referred, the discriminating physician can never be at a loss to distinguish one disease from the other. The remote cause of milk-sickness being buried in obscurity has no doubt induced many to question its existence. Such persons might as well doubt that man has a spleen, because the most acute anatomists have not yet discovered its use.

*July* 11*th*, 1840.

